# Society Meetings

**Published:** 1917-06

**Authors:** 


### SOCIETY MEETINGS

**Brief reports and announcements of meetings of societies of Western New York, and those of general scope, are requested from Secretaries. Copy should be on hand the fifteenth of the month. Full transactions will be published at cost of composition.**

---

**Medical Society of the County of Erie.** Regular meeting in the Buffalo Medical College, April 16th. In the absence of the President, Vice-president Dr. George F. Cott presided. The minutes of the regular meeting held in February and also the minutes of the Council were read and adopted.

The following were reinstated: Dr. George Schaefer, 967 Jefferson St.; Dr. Bruce L. D. Cook, 819 Jefferson St.; Dr. George B. Stocker, 855 Genesee St.

The following new members were elected: Dr. Charles B. Handel, 19 East Genesee St.; Dr. John A. P. Millet, 520 Franklin St.; Dr. Byron D. Bowen, 84 Elmwood Ave.; Dr. M. Carlton Vaughan, 78 Brayton St.; Dr. Clifford Rowell, 224 Massachusetts Ave.; Dr. Adam R. Johnson, 478 Delaware Ave.; Dr. Jane R. Breese, 441 Franklin St.; Dr. William P. Clothier, 1126 Main St.; Dr. Alexander Mulki, 41 Cedar St.; Dr. John A. Metzen, 1335 West Ave.

The Society instructed its delegates to the State Society to oppose any move which would change the ratio of representation in the State Society.

It also instructed its delegates to oppose the candidacy of any person who had formerly been active as a proponent of the Mills Bill.

The speakers of the evening were to have been Dr. Thomas W. Salmon Medical Director of the National Committee for Mental Hygiene and Dr. Ethan A. Nevin, Superintendent of the State Custodial Asylum for Feebleminded Women, Newark, N. Y. At the last moment, however, Dr. Salmon, who is on the Medical Reserve List of the War Department received orders, which made it impossible for him to come to Buffalo, and he therefore requested Dr. Frankwood E. Williams, Association Medical Director of the National Committee for Mental Hygiene, Editor of the new magazine on Mental Hygiene, and former Secretary of the Massachusetts Mental Hygiene Society to attend in his place.

Dr. Nevin accompanied his remarks by stereopticon slides and motion picture reels.

A hearty vote of thanks was tendered to each of the speakers after which adjournment was made to the College Library where a collation was served.

FRANKLIN GRAM, Secretary.

---

**The American Gastro-Enterologic Assn.** held its 20th annual meeting in Atlantic City, Apr. 30 and May 1, under the presidency of Dr. Wm. Garry Morgan of Washington.

---

**The Alienists and Neurologists** will meet under the auspices of the Chicago Medical Society, in Chicago, July 10-12. The program will include discussions of state hospital architecture, custody, special hospitals for research and prevention, colonies, etc., as well as papers on diseases. There are no fees of any kind. Those interested should address the Secretary, Dr. Bayard Holmes, 30 N. Michigan Ave., Chicago.

---

**The Women's Medical Society of N. Y. State** met in Utica, Apr. 23, before the Medical Society of the State of N. Y., under the presidency of Dr. Eveline P. Ballintine, of Rochester. It concluded with a banquet, Dr. Maud J. Frye of Buffalo acting as toastmistress.

---

**The Elmira Academy of Medicine** held its regular meeting May 2. Dr. H. W. Fudge reported a case of Acute Double Suppurative Parotitis; and Dr. Charles Haase made a report on the proceedings of the State Society. Pres. Dr. R. B. Howland, Sec. Dr. O. J. Bowman.

**The Alumni Assn. of the Medical Dept., University of Buffalo,** holds its 42d annual meeting May 31-June 1. Last year, owing largely to military duties, the committee was unable to prepare the transactions for publication as customary but we hope that, in spite of the increased urgency of political conditions, the publication will be resumed this summer.

---

**The Rochester Academy of Medicine, Section I,** met May 9. Dr. James P. Brady read a paper on Clinical Signs and Symptoms of Hepatic Disease; Dr. George W. O'Grady on Functional Tests of Hepatic Function and Disease.

---

**The Buffalo Academy of Medicine** has held the following meetings since last reported: Apr. 25, Section of Pathology, Army and Naval Service Opportunities and Duties, Dr. Harry Lee Brown, Surgeon, U. S. N.

May 2, Section of Surgery, Treatment of Compound Fractures, Dr. Frank W. McGuire, discussion opened by Dr. Harry Trick.

May 9, Section of Medicine, Phlyctenular Eye Affections and Tuberculin, Dr. H. H. Glosser.

May 16, Section of Obstetrics, Adeno-cystoma of the Ovaries, with microscope demonstration, Dr. Chas. A. Bentz. (Note: The newly elected officers of the Academy will be announced together, in a later issue).

---

**The Pathological Society** of Rochester, held a meeting May 16. Dr. E. W. Jackson gave a paper on "How Can We Best Tell the Condition of the Heart Muscle?" Pres. Dr. H. J. Vary; Sec. Dr. G. H. Gage.

---

**The Gross Medical Club** held its meeting at the Hotel Lenox, May 18, being entertained by Dr. Grover W. Wende; in the absence of President King, Dr. B. E. Smith, Vice-President, occupied the chair.

On motion of Dr. J. Henry Dowd it was unanimously voted to donate $50 to the Red Cross to be used for a bed in the base hospital.

Dr. Benj. Gipple of Alden read a paper, "Biological Therapeutics," discussed most vigorously by all, the unanimous opinion being that, "Some few had virtue, but many were humbugs foisted on a credulous body of men who have licenses to treat sick people."

Several very important cases were reported by Drs. Cott, King, Bennett, Wende and others. The banquet following was a credit in every way to this well known hotel.

